# Non random distribution of child undernutrition in Ethiopia: spatial analysis from the 2011 Ethiopia demographic and health survey

**DOI:** 10.1186/s12939-016-0480-z

**Published:** 2016-12-03

**Authors:** Zewdie Aderaw Alemu, Ahmed Ali Ahmed, Alemayehu Worku Yalew, Belay Simanie Birhanu

**Affiliations:** 1 Public Health Department, College of Health Sciences, Debre Markos University, P.O. Box 269, Debre Markos, Ethiopia; 2 School of Public Health, College of Health Sciences, Addis Ababa University, P.O.Box 14 575, Addis Ababa, Ethiopia; 3 Center for Environment and Development, College of Developmental Studies, Addis Ababa University, P. O. Box 56649, Addis Ababa, Ethiopia

**Keywords:** Child undernutrition, nonrandom, Ethiopia Demographic and Health Survey, Spatial, SaTScan, Arc GIS, Ethiopia

## Abstract

**Background:**

Child undernutrition showed geographical inequalities due to variations in contextual determinants from area to area which indicates that location is an important factor in child undernutrition. However, there are limited studies on spatial epidemiology of child undernutrition in Ethiopia. This study was aimed to identify the SaTScan spatial clusters of child undernutrition in Ethiopia.

**Methods:**

Nutritional indices of children (0–59 months) with Global Positioning System (GPS) location data were accessed from the 2011 Ethiopia Demographic and Health Survey (EDHS) after getting permission from the MEASURES Demographic and Health Survey (DHS) Program. The Bernoulli Model was fitted using SaTScan™ software, version 9.4. for SaTScan cluster analysis. Log Likelihood Ratio (LLR) test was used for each SaTScan cluster and size of the scanning SaTScan cluster to test the alternative hypothesis that there is an elevated risk within the SaTScan cluster compared to outside the SaTScan cluster. Less than 0.05 for LLR was considered as statistically significant level.

**Results:**

The SaTScan spatial analysis result detected Liben, Afder and Borena administrative zones around the South East Ethiopia as the most likely primary spatial SaTScan clusters (LLR = 28.98, *p* < 0.001) for wasting. In the Northern, Middle, North East and North West areas of Ethiopia particularly from all administrative zones of Amhara, Tigray, Afar, Ben. Gumz regional states and East Welega and North Showa zones from Oromiya Regional State (LLR = 60.27, *p* < 0.0001) were detected as the most likely primary SaTScan clusters for child underweight. Also in the Northern, Middle, North East and North West areas of all administrative zones of Tigray, Amhara, Ben. Gumz and Afar regional states and West and North Showa and East Welega from Oromiya Regional States (LLR = 97.28, *P* < 0.0001) were primary SaTScan clusters for child stunting.

**Conclusion:**

The study showed geographical variability of child stunting, underweight and wasting in the Country which demands risk based local nutritional interventions. Further study will be important to assess the temporal nature of the problem and to identify community level factors that create this spatial variation.

## Background

Malnutrition refers to any disorder of nutrition whether it is due to dietary deficiency or to excess diet which can result from an imbalance between the needs of the body and intake of nutrients [1]. The common malnutrition type in low income countries, including Ethiopia is undernutrition and Food and Agricultural Organization (FAO) defined under nutrition as the proportion of people whose dietary energy consumption is continuously below a minimum dietary energy requirement for maintaining a healthy life and carrying out light physical activity with an acceptable minimum body weight for height [2]. Child undernutrition can be characterized by low height for age, low weight for height and low weight for age. Stunting is defined a low height-for-age at < −2 Standard Deviation (SD) of the median value of the WHO international growth reference and underweight is defined as low weight-for-age at < −2 Standard Deviation (SD) of median value of the WHO international growth reference. Wasting refers to low weight-for-height at < −2 Standard Deviation (SD) of median value of the WHO international growth reference [3].

Undernutrition affects growth and development of infants [4–6], cognitive and academic performance of school children and psychosocial interactions of the society, causes anxiety, depression and other symptoms of common mental illness [7–10]. Malnutrition, including child undernutrition in human beings specially in children prevents from reaching their full physical and mental potential which leads them to delay in their physical growth and motor development, attention deficit disorder, impaired school performance, reduced language development performance, learning abilities, decreased IQ scores, memory deficiency, reduced problem-solving abilities, lower intellectual quotient (IQ), leads to greater behavioral problems and deficient social skills and susceptibility to contracting diseases [1, 2].

The under 5 years old children global prevalence of underweight showed geographical disparity between developed (2.4%) and developing (17.4%) countries [11]. The problem is higher in Africa (17.7%) and more severe in East Africa (19.3%) and Sub Saharan Africa (SSA) (21.4%) [11]. Also the global prevalence (8%) of under 5 years old child wasting showed variation between developed (1.7%) and developing (8.8%) countries. The magnitude is higher in Africa (8.5%) and SSA (9.4%) compared to the global level prevalence [11].

World Health Organization (WHO) in its 2005 Bulletin indicated that the occurrence of underweight children in Africa showed a clear disparity among countries [11]. The problem was higher in northern Nigeria, and adjacent Niger and large areas of Ethiopia, Sudan and Eretria [12].

According to the Ethiopia Demographic and Health Survey (EDHS) 2011 Report, the overall prevalence of child stunting, underweight and wasting was 44%, 29% and 10%, respectively and there was a wide variation in child nutritional status among regional states [13].

The issue of undernutrition is a complex phenomena and spatial analysis offers a new potential to get a better understanding of this complexity in public health research [14]. Spatial analysis is very important to allocate scarce resources to the most affected areas, to plan interventions effectively and to design cost effective intervention strategies [14]. As a result, epidemiologists are gradually incorporating spatial analysis in health including malnutrition [14].

Different spatial analysis studies have been done in SSA by aggregating the undernutrition indicators either to national or regional levels and efforts have been made to visualize the spatial distribution of child undernutrition using a map [15]. The methodological strength of those studies ranges from the mapping of malnutrition to the advanced spatial statistical analysis to identify the impact of environmental and other geographical factors on malnutrition in SSA [15]. As a result, researchers, policymakers, and program managers have recognized location as an important factor in population and health outcomes. This is especially true for child undernutrition that can be influenced by geographical, cultural and environmental factors such as, altitude, rainfall, soil fertility, crop productivity, population density and infectious diseases distribution [15].

However, in Ethiopia there is no study on spatial epidemiology of child undernutrition using SaTScan analysis except one study which focused only on stunting indicator and found that the non random distribution of child stunting with high clustering in the northern highlands of Ethiopia [16]. Therefore, this analysis was designed to identify the SaTScan clusters of child stunting, underweight and wasting which is very important to identify and focus on most vulnerable groups, to plan interventions and to allocate scarce resources effectively.

## Methods

### Study area and setting

Ethiopia is situated in the Horn of Africa, between 3 and 15 degrees north latitude and 33 and 48 degrees east longitude. It is a country with great geographical diversity; its topographic features range from the highest peak at Ras Dashen, which is 4,550 meters above sea level, down to the Afar Depression at 110 meters below sea level. As the country is located within the tropics, its physical conditions and variations in altitude have resulted in great diversity of terrain, climate, soil, flora, and fauna [13].

In Ethiopia, the mean maximum and minimum temperatures vary spatially and temporally. Generally, the mean maximum temperature is higher from March to May and the mean minimum temperature is lower from November to December compared to other months [13]. Ethiopia’s mean annual distribution of rainfall is influenced by the direction of both westerly and southeasterly winds. Thus, in Ethiopia the general pattern of annual rainfall distribution remains seasonal, varying in amount, space, and time [13].

### Study design and sample

From the 2011 EDHS, 9893 children aged from 0 to 59 months, 9512 children in 571 EDHS clusters were considered for analysis [13]. A total of 381 children were excluded from the analysis since of either their nutritional indices were flagged or geographical location data were not available.

### Data source and measurements

Administratively, the 9 regions and two town administratives in Ethiopia are divided into Zones, and Zones, into administrative units known Weredas. Each Wereda is further subdivided into the lowest administrative units, called Kebele. During the 2007 census, each kebele was subdivided into census enumeration areas (EAs), which were convenient for the implementation of the census [13].

The 2011 EDHS sample was selected based on the above classification using a stratified, two-stage cluster design, and EAs were the sampling units for the first stage and Households comprised of the second stage of sampling [13].

The EDHS team collected data on height and weight from under five children in all selected households. Weight measurements were obtained using light weight, SECA mother-infant scales with a digital screen, designed and manufactured under the guidance of United Nations International Children Emergency Fund (UNICEF) [13].

Height measurements were carried out using a measuring board. Children younger than 24 months were measured for height, while lying down, and older children, while standing [13]. For all indices of child undernutrition, Z score < −2 SD from the median of the WHO reference population was considered as undernourished [13]. Geo reference coordinates were collected at EDHS cluster level using hand-held GPS [13].

The EDHS team cleaned the data and calculated the Z score for all child nutritional status indices. Child nutritional status data from the 2011 EDHS clusters were characterized by unique latitude and longitude location coordinates. Then, the EDHS cluster nutritional data and location file data were cross linked using the Arc GIS 10 in making maps [17].

The existing nutritional indices Z score was used by the research team to determine child nutritional status. A shape file with district boundaries and polygon shapes were obtained from the Central Statistical Agency of Ethiopia (CSA) [13].

### Data management and analysis

The number of undernourished and non undernourished children in each location had Bernoulli distribution. In such type of data, the analysis was done using Bernoulli model from SaTScan™ software, version 9.4 which requires undernourished (as cases) and non undernourished (as controls) children represented by 0/1 variable [18]. Also the Bernoulli model requires information about the location of a set of undernourished and non undernourished children provided to SaTScan [18].

SaTScan software used a circular window moved systematically throughout the study area to identify significant SaTScan clustering of child undernutrition and centered on each of a number of possible locations throughout the study EDHS clusters and for each location. SaTScan cluster analysis was performed with maximum spatial cluster size of <50% of the population at risk using the default. This fifty percent was specified as the upper limit, which allowed both small and large SaTScan clusters to be detected and ignored SaTScan clusters that contained more than 50% of the population. Log Likelihood Ratio (LLR) test was used to test the hypothesis that there were elevated undernourished children compared with the distribution outside the moving SaTScan cluster.

The window sizes and locations with the maximum likelihood were defined as the most likely SaTScan cluster (s) and it is the least likely to have occurred by chance. The LLR test was used for each location and size of the scanning SaTScan cluster to test the alternative hypothesis that there is an elevated risk within the SaTScan cluster compared to outside the SaTScan cluster [18].

Monte Carlo replications of the dataset determined the distribution and *p*-value of the most likely and secondary SaTScan clusters. The aim was to detect geographical locations with high numbers of undernourished children. A standard of ‘no geographical overlap’ was used to report secondary SaTScan clusters. The *P*-value was created using the combination of approximation and used 999 replications of Monte Carlo which is recommended one for the Bernoulli modeling [18].

## Results

Majority of the study participants (86.8%) were from the rural area. From the total children included in the study, 49.2% were males.

### Spatial epidemiology of child wasting

**Fig. 1:**
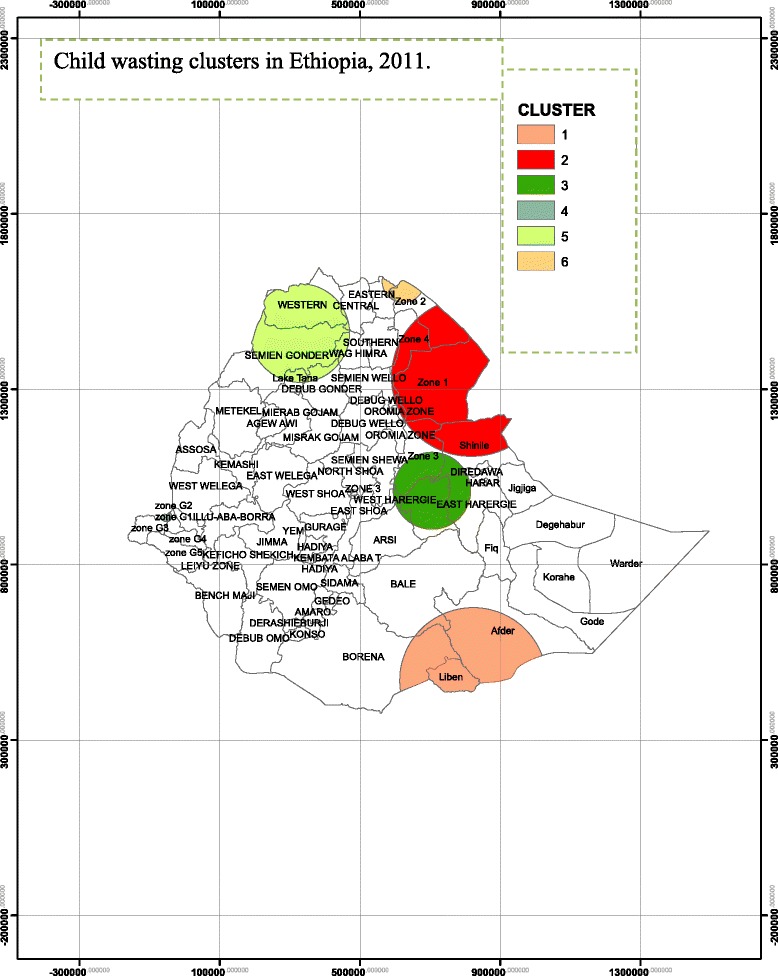
Spatial Distribution of child wasting in Ethiopia, EDHS 2011

A total of 1201 (12.6%) under five children were wasted. As indicated in the Fig. 1 below, a total of six SaTScan clusters were identified in the order of child wasting severity from the SaTScan spatial analysis, but only four of them were statistically significant circular windows which means that the severity is higher inside the circular window compared to outside the SaTScan clusters.

The SaTScan spatial analysis result detected the most likely primary spatial SaTScan clusters (LLR = 28.98, *p* < 0.001) for wasting around South East Ethiopia specifically Liben, Afder and Borena administrative zones.

Also the most likely secondary SaTScan clusters (LLR = 24.8, *P* < 0.0001) for child wasting was observed from East Ethiopia of Shinile from Somali Regional State and Zone 1 and Zone 4 administrative zones from Afar Regional State. The SaTScan spatial analysis also indicated statistically significant most likely secondary SaTScan clusters at the 3rd and 4th stage. In the Eastern part of Ethiopia, specifically from East and West Harargie from Oromiya Regional State and Zone 3 from Afar Regional State were identified as containing the location of the most likely SaTScan clusters at the third stage (LLR = 13.68, *P* = 0.001). In the fourth stage, SaTScan clusters were identified in South West Ethiopia of Gambella Regional State (LLR = 10.77, *P* = 0.012).

Other SaTScan clusters at the 5^th^ (LLR =7.74, *P* = 0.151) and 6^th^ (LLR = 6.64, *P* = 0.37) SaTScan cluster locations did not show statistically significant difference inside the SaTScan cluster compared to outside the SaTScan cluster in terms of child wasting. Table 1 above summarizes the 6 identified SaTScan clusters using SaTScan spatial analysis.

**Table 1:** SaTScan spatial analysis of child (0–59 months) wasting in Ethiopia from EDHS 2011 data (*n* = 9512)

Cluster	Zones in the cluster	Coordinate/radius	LLR^a^	*P* value
1	Liben, Afder and Borena	4.2400 N, 41.9060E/208.94KM	28.98	<0.0001
2	Shinile, Zone 1 and zone 4	12.0559 N,41,9155E/227.0KM	24.18	<0.0001
3	East and West Harargie, 3one 3	9.1376 N,40.8658E/110.OKM	13.68	0.001
4	Ben. Gumz special zone	8.4495 N,33,9925E/15.4KM	10.77	0.012
5	Western, North and south Gondar	13.2022 N,37.4207/141.33KM	7.74	0.151
6	Eastern and Zone 4	14,5352 N,40.1473E/60.33KM	6.64	0.37

^a^LLR refers to loglikelihood ratio

### Spatial epidemiology of child underweight

**Fig. 2:**
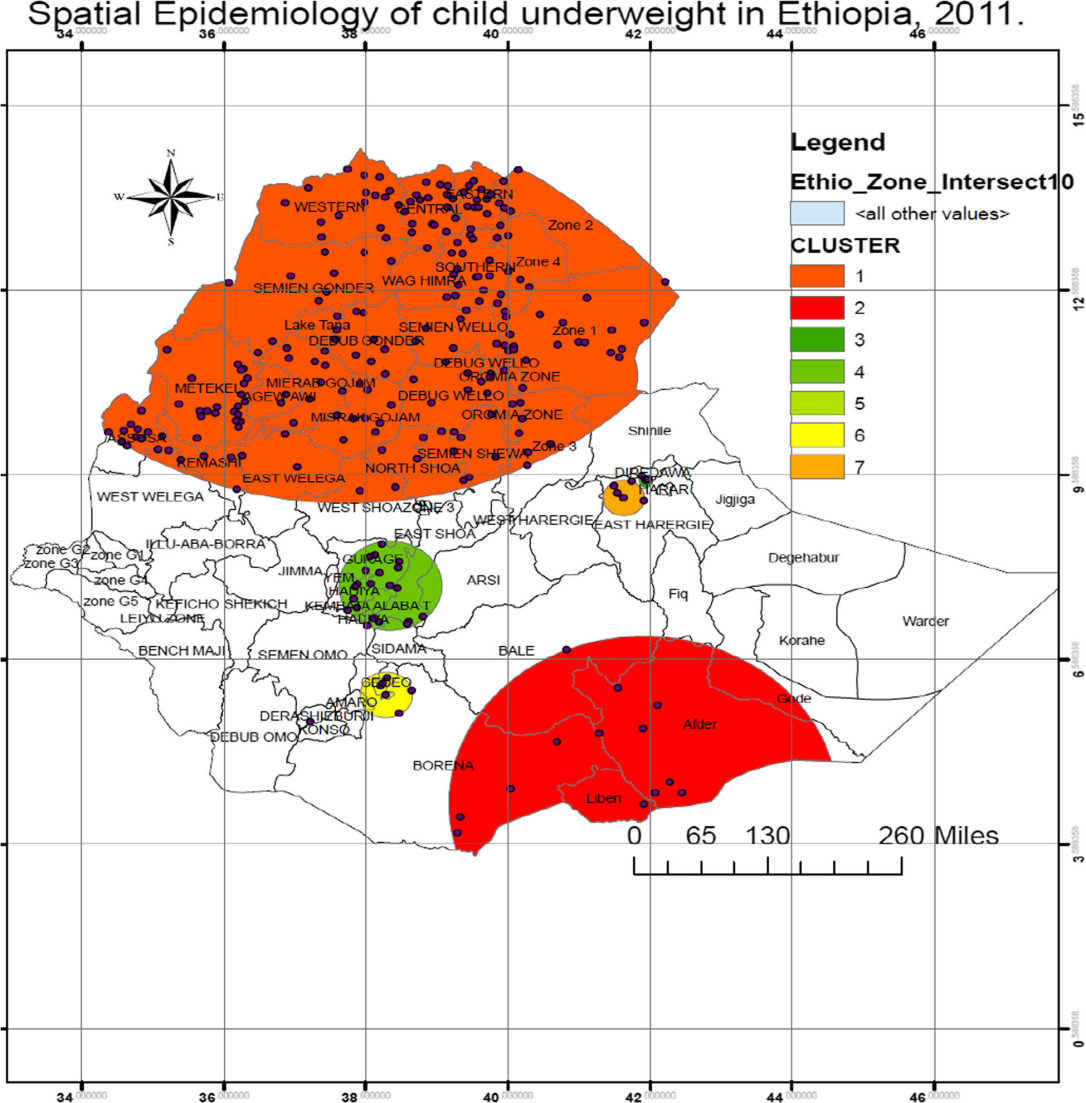
Spatial Epidemiology of child underweight in Ethiopia, EDHS 2011

A total of 9638 under five children were considered and 2977 (30.88%) under five children were underweight and 6661 (69.11%) were not underweight children. The SaTScan spatial analysis identified a total of 7 SaTScan clusters as hotspot areas for child underweight in the order of severity. Figure 2 above indicates the geographical distribution of child underweight SaTScan clusters in the order of severity.

The most likely primary SaTScan clusters were detected in the Northern, Middle, North east and North west areas of Ethiopia, particularly from all administrative zones of Amhara, Tigray, Affar, Ben. Gumz regional state administrative zones and East Welega and North Showa administrative zones, from Oromiya Regional State (LLR = 60.27, *p* < 0.0001).

Also most likely secondary SaTScan clusters were detected in Southeast areas of Ethiopia particularly from Liben, Afder, Borena and Gode administrative zones (LLR = 13.65, *P* = 0.001). In the third location, the SaTScan spatial analysis identified SaTScan clusters in Southern and central Ethiopia, particularly from East showa, Guragie, Kembata Alaba T, Hadiya, Sidama and Arsi zones (LLR = 10.03, *P* = 0.024). The rest four SaTScan clusters identified during the spatial analysis inside the SaTScan clusters were not significantly different from outside the SaTScan clusters in terms of child underweight. The details of the SaTScan spatial analysis result is presented in the Table 2 below.

**Table 2:** SaTScan spatial analysis of child (0.–59 months) underweight in Ethiopia from EDHS 2011 data (*n* = 9512)

Cluster	Zones in the cluster	Coordinate/radius	LLR	*P* value
1	All zones from Tigray, Amhara Ben. Gumz, and Afar and East Wolega and North Showa from Oromiya	14.5490 N, 37.7590E/602.32KM	60.27	<0.0001
2	Liben, Afder, Borena and Gode	4.2400 N, 41.9060E/303.23KM	13.65	0.001
3	Harar	9.4935 N, 419374/10.68KM	10.03	0.024
4	Gurage, Hadiya and Alaba T	7.7850 N, 38.3423E/80.44KM	6.69	0.330
5	Derashie	5.5705 N, 37.2128E/OKM	6.61	0.363
6	Gedeo	6.0119 N, 38.2839E/41.31KM	6.01	0.874
7	East Harargie	9.2141 N, 41.6246E/32.19KM	9.21	0.992

### Spatial epidemiology of child stunting

**Fig. 3:**
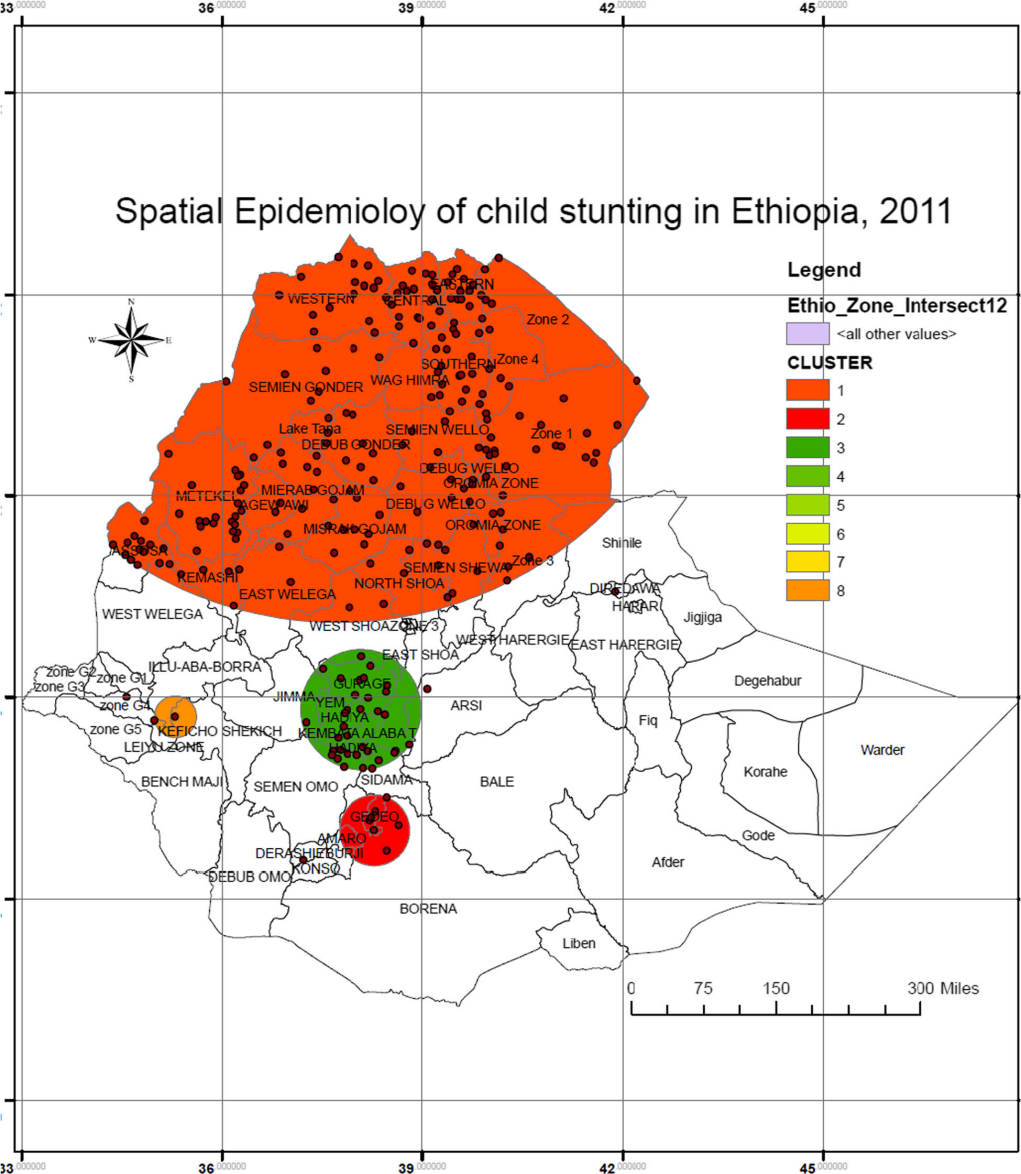
Spatial Epidemiology of child stunting in Ethiopia, EDHS 2011

A total of 9572 children were considered for the analysis and 4149 (43.3%) of them were stunted. The SaTScan spatial analysis detected a total of 8 SaTScan clusters for child stunting. The geographical distribution of child stunting SaTScan clusters in Ethiopia is indicated in Fig. 3 above.

As indicated in Table 3 below, from the spatial SaTScan analysis, the most likely primary SaTScan clusters were detected around Northern, Middle, North east and North west areas of Ethiopia particularly from all administrative zones of Tigray, Amhara, Ben. Gumz and Affar regional states and West and North Showa and East Welega from Oromiya regional states (LLR = 97.28, *P* < 0.0001).

**Table 3:** SaTScan spatial analysis of child (0–59 months) stunting in Ethiopia from 2011 EDHS data (*n* = 9512)

Cluster	Zones in the cluster	Coordinate/radius	LLR	*P* value
1	All zones from Tiray, Amhara, Affar Ben. Gumz. and West and North Showa and East Wollega from Oromiya region	14.549 N, 37.7358E/604.20KM	97.28	<0.0001
2	Borena and Gedeo Zone	6.0119 N,38.2	9.13	0.06
3	Guragie, Hadiya, Kemebata Alaba T, Yem, North omo, Jimma, Sidama and East Showa zones	7.8144 N, 38.0783E/	5.67	0.682
4	Derashie	5.5705 N, 37.2128E/99.70KM	4.53	0.954
5	Dire Dawa	9.5725 N, 41.8792E/0KM	4.45	0.959
6	Zone 4 from Gambella	8.0017 N, 34.5765E/0KM	4.14	0.981
7	Arsi Zone	8.1210 N, 39.0798E/0KM	3.90	0.992
8	Kefich shekicho and Gambella special zones	7.7087 N, 35.3035E/34.71KM	3.71	0.997

The SaTScan spatial analysis result indicated the secondary most likely SaTScan clusters from Southern Ethiopia specifically from Borena and Gedeo Zones (LLR = 9.13, *p* = 0.06). The rest of the SaTScan clusters identified using SaTScan spatial analysis were not statistically significant difference between inside and outside the SaTScan clusters.

## Discussion

The Government of Ethiopia recognized the need for providing equitable access to promotive, preventive, and selected curative health services to its people, and launched the health extension program (HEP) in 2003 to facilitate achieving universal access to Primary Health Care and accelerate the country’s progress, including reducing hunger and child mortality [18]. Child nutrition is one of the seven major components of the health extension packages which is the responsibility of the Health Extension Workers [19].

Due to the above efforts, Ethiopia has made a considerable progress in several dimensions, including reducing the burden of child malnutrition [3]. In the country, food insecurity remain a big challenge and over 30% of the population are below the food poverty line, unable to afford the minimum caloric intake for a healthy and active life [15]. The magnitude of the problem is not uniform throughout the country due to different reasons [13]. In Ethiopia, the government Health Policy and Health Sector Transformation plan give emphasis to bring equity to reduce the disparity [16, 20, 21].

According to the UNICEF conceptual framework, child nutritional status is affected by food, health and care related factors and those are again affected by social, economic and political factors. The combination and relative importance of these factors differ from country to country and even within a country from one area to another [22]. Those factors may differ by geographical location which may lead to variation in child nutritional status [21]. This SaTScan spatial analysis of the 2011 EDHS indicate that child malnutrition status in Ethiopia show statistically significant geographical variation in all nutritional status indicators (stunting, underweight and wasting). Studies elsewhere as well as in the country supported the non random distribution of child malnutrition. Studies from West Africa [23], SSA [24], Nigeria [25] and Ethiopia [16, 26] indicated a clear spatial pattern in child under- nutrition across different geographical locations [16, 25–27].

From this 2011 EDHS data spatial statistics analysis, the most likely primary SaTScan clusters (hotspot areas) were detected in different geographical locations using all the three protein energy malnutrition indicators. The primary most likely SaTScan clusters for child wasting were detected in the southern parts of the country, particularly from Liben, Afder and Borena administrative zones. The geographical location of environmental, socioeconomic, cultural and health service utilization related determinants may cause those child wasting variations. Patterns of child malnutrition distribution suggest that local area (SaTScan cluster) characteristics as well as local administrative circumstances play a role in the causation or prevention of under nutrition. Those local and regional factors may be related to agronomic or climatic conditions and pattern, of major diseases [28] and understanding the contexts of child malnutrition that leads to spatial inequality is critical to meet the needs of most vulnerable people for child malnutrition [21].

The prevalence of wasting, underweight and stunting declined from 12% to 10%, 41% to 29% for underweight and 58% to 44.4% for stunting between 2000 and 2011, respectively. But still, the magnitude is categorized as ‘serious’ by WHO cut offs [29]. For both child underweight and stunting, this SaTScan spatial analysis detected most likely primary clusters in Northern, Middle, North east and North west areas of Ethiopia, particularly from Tigray, Amhara, Benishangul Gumuz and Afar regional states and North Showa and East Wollega administrative zones in Oromiya Regional State.

A child who lives in the above mentioned geographical locations has a probability of high risk for undernutrition compared with those who live outside the SaTScan cluster. This spatial inequality may be related with the populations in the regions of Amhara, Tigray, Afar and Benishangul Gumuz, mainly those who live in rural areas where the problem is more prevalent compared with urban centers [29]. Also it is reported that the four region have higher rates of food poverty, over 30% of households in Tigray and Amhara are below the food poverty line and more than 25% of households in Afar and Benishangul Gumuz [29]. A large proportion of households consume less than 2550 kilocalories per adult equivalent per day particularly in Amhara Region (37%) [29].

The spatial pattern of child wasting is different from that of stunting and underweight, which might be related to it which is an acute measure of nutritional status and more sensitive to seasonal variability. Unlike stunting, child wasting is not a recommended indicator to see spatial patterns of child undernutrition since it varies seasonally [30].

This study tried to see all indices of child undernutrition. The result can be used for policy makers since effective stewardship of health programs including nutrition requires approaches that precisely target resources and interventions to meet population needs; one approach involves the use of geographic data modeling [30]. Also this study has tried to assess the spatial epidemiology of child undernutrition in Ethiopia which was not common epidemiological investigation in nutritional related researches previously. However, the study is not free from limitations. The study did not do an adjustment for covariates during estimation of the spatial epidemiology of child malnutrition. The missed data may affect the true estimates of the analysis. Also there may be false inclusion and exclusion of SaTScan clusters that were not similar in shape to the scanning SaTScan cluster could produce errors which means it cannot detect holes in SaTScan clusters.

## Conclusion

This study showed geographical variability of stunting, underweight and wasting in the country. Therefore, nutrition intervention programmers should give emphasis to the identified hotspot areas to reduce child malnutrition by developing local based intervention strategies. A further study on the causes of malnutrition should be conducted in the identified hotspot areas to design local specific interventions.

### Policy implications

To improve the overall national nutritional status, decision makers should give emphasis to the identified SaTScan clusters through developing local interventional strategies. The country is striving to achieve both sustainable development goals and the Ethiopian SQOTA declaration to make zero stunting by 2030 through targeting interventions to the most risky areas. So this study result will help to allocate resources more efficiently. Different governmental and nongovernmental organizations that are working on nutrition can benefit from the research to bring equity by giving emphasis to the most affected areas.
